# Multivariate Analysis of Risk Factors for Atlantoaxial Osteoarthritis: A Retrospective Cohort Study on Ligament Ossification, Joint Degeneration, and Muscle Fatty Infiltration

**DOI:** 10.1111/os.70125

**Published:** 2025-09-01

**Authors:** Shuqing Jin, Shuhao Zhang, Yuxiao Zhu, Yan Chen, Yiting Tu, Yurui Wu, Siyu Hu, Chen Xiang, Xiangyang Wang

**Affiliations:** ^1^ Department of Orthopaedics The Second Affiliated Hospital and Yuying Children's Hospital of Wenzhou Medical University Wenzhou Zhejiang Province China; ^2^ Key Laboratory of Orthopaedics of Zhejiang Province Wenzhou Zhejiang Province China; ^3^ The Second School of Medicine Wenzhou Medical University Wenzhou Zhejiang Province China

**Keywords:** atlantoaxial osteoarthritis, fatty infiltration, inter‐atlanto‐occipitalligament, the obliquus capitis inferior muscles, uncovertebral joint degeneration

## Abstract

**Objective:**

Atlantoaxial osteoarthritis (AAOA) cause occipitocervical and retroauricular pain and cervical rotation disorder. Few studies have focused on the relationship between cervical spine structure and the prevalence of AAOA in China. This study aimed to investigate whether the inter‐atlanto‐occipital ligament ossification, uncovertebral joint degeneration and fat infiltration (FI) in the obliquus capitis inferior (OCI) muscles are associated with atlantoaxial arthritis, and to explore other potential risk factors in a clinical cohort from Eastern China.

**Methods:**

We analyzed CT images of the upper cervical spine from 1021 adult trauma patients scanned at our hospital between January 1, 2014, and July 1, 2024. Atlantoaxial osteoarthritis and uncovertebral joint degeneration were categorized as none‐to‐mild (no osteoarthritis) or moderate‐to‐severe (osteoarthritis present). Ossification of the inter‐atlanto‐occipital ligament was graded 0–3 based on its extent. Risk factors for atlantoaxial osteoarthritis were identified using univariate and multivariable logistic regression analyses. Among these patients, 381 underwent cervical MRI, and we assessed fat infiltration (FI) in the inferior oblique muscles, classifying it into quartiles: mild (8.51%–18.49%), moderate (18.67%–31.56%), and severe (31.88%–46.22%). Multivariate regression analysis was then performed to explore the relationship between FI severity and the incidence of AAOA.

**Results:**

The study group consisted of 59.4% men, with a mean age of 50.18 ± 17.23 years, and an AAOA prevalence of 11.6%. In the primary multivariable logistic regression analysis, the following factors were independently associated with AAOA: age ≥ 50 years (OR 30.48, *p* < 0.001), inter‐atlanto‐occipital ligament ossification (OR 1.59, *p* = 0.033), female sex (OR 2.54, *p* < 0.001), and uncovertebral joint degeneration in the lower cervical spine (OR 2.38, *p* < 0.001). In a separate multivariate logistic regression analysis that specifically included the degree of fatty infiltration in the inferior oblique muscles, it was found that greater fatty infiltration was also significantly associated with an increased risk of AAOA (OR 3.52, *p* < 0.001).

**Conclusions:**

Age over 50 years, inter‐atlanto‐occipital ligament ossification, female sex, and uncovertebral joint degeneration are significant factors associated with atlantoaxial osteoarthritis. Severe fatty infiltration of the inferior oblique muscles may also be a potential risk factor. Delayed diagnosis and treatment may be prevented by prioritizing these risk factors.

## Graphical abstract

This study investigated risk factors for atlantoaxial osteoarthritis (AAOA), including inter‐atlanto‐occipital ligament ossification, uncovertebral joint degeneration, and fatty infiltration (FI) of the obliquus capitis inferior (OCI) muscles. A retrospective analysis of 1021 adult patients identified significant associations between AAOA and age ≥ 50 years, female sex, ligament ossification, uncovertebral joint degeneration, and severe FI. These findings highlight key structural changes in the cervical spine linked to AAOA prevalence, aiding in early diagnosis and prevention.

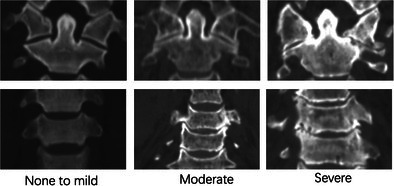

## 1 Introduction

Atlantoaxial osteoarthritis (AAOA) is characterized by a syndrome that induces pain in the upper neck, suboccipital, occipitocervical, occipital, or postauricular regions [1]. Ehni and Benner have documented that this pain is typically deep, boring, or aching in nature and is associated with restricted movement [2].

Previous research has indicated that the prevalence of AAOA ranges from 4% to 9%, but data specific to the Chinese population is lacking [3, 4, 5]. Studies have identified factors such as female sex, advanced age, occupations involving head loading, and calcific synovitis as potential contributors to the development of AAOA [6, 7]. However, limited attention has been given to the role of craniocervical ligaments, uncovertebral joints, and cervical muscles, despite their essential function in maintaining cervical spine stability. Understanding the influence of these structures may provide further insights into the pathogenesis of AAOA.

Various ligaments and membranes are essential for maintaining stability at the craniocervical junction [8]. Lesions in this region can lead to severe clinical outcomes, including craniocervical dislocation [9, 10, 11]. Liu et al. reported an unidentified ossification in this region that may be associated with the inter‐atlanto‐occipital ligament, called the capped dens sign, and in their series, more than one‐third of patients with cervical spondylosis demonstrated CT evidence of ossification in this region; however, the relationship between this ossification and AAOA has not yet been studied [12]. Given the critical role of ligamentous structures in maintaining stability at the craniocervical junction and the serious clinical implications of lesions in this area, further investigation into the potential associations between such ossifications and conditions like AAOA is warranted [13].

The uncinate processes and uncovertebral joints are unique to the cervical spine and play a key role in guiding vertebral motion during head and neck movement [14], [15] Degeneration of these joints, commonly seen in middle‐aged and elderly individuals, is linked to conditions such as cervical spondylosis and radiculopathy [16]. Given the crucial role of the uncovertebral joints in cervical mechanics, degeneration in this region may contribute to the development of other cervical pathologies, including atlantoaxial osteoarthritis (AAOA).

The suboccipital muscles are essential for maintaining the stability of the craniocervical junction [17]. The obliquus capitis inferior (OCI) muscle, a key suboccipital muscle, lies deep between the spinous process of C2 and the transverse process of C1, aiding in head rotation [18]. Fatty infiltration (FI) of skeletal muscles is a common degenerative change associated with aging and reduced muscle function. This process, characterized by the replacement of muscle fibers with fat, can lead to diminished muscle strength and compromised joint stability [19, 20]. However, existing research has paid little attention to the fatty infiltration of the OCI muscle. Given the muscle's role in stabilizing the atlantoaxial joint, we hypothesize that FI of the OCI may also be associated with the development of atlantoaxial osteoarthritis (AAOA).

So we conducted a retrospective study, and the purposes of this study are threefold: (1) to determine the prevalence of atlantoaxial osteoarthritis (AAOA) in a clinical cohort from Eastern China; (2) to investigate whether ossification of the inter‐atlanto‐occipital ligament, uncovertebral joint degeneration in the lower cervical spine, and other cervical degenerative changes are potential risk factors for AAOA; and (3) to assess age‐related changes in the obliquus capitis inferior muscle and explore whether severe fatty infiltration of this muscle is a potential risk factor for AAOA.

## 2 Method

### 2.1 Ethics Approval

This study was approved by the Ethics Review Board of our institution (MR‐33‐25‐000548), with the requirement for patient informed consent waived due to its retrospective design.

### 2.2 Patients

Eligible participants were adult patients admitted to our emergency and critical care center between January 1, 2014, and July 1, 2024, who underwent cervical spine CT as part of their diagnostic evaluation (*n* = 1831).

Inclusion criteria were as follows:

- Age 18 years or older;
- Underwent cervical spine MRI and CT imaging between January 2019 and December 2022;
- Complete clinical and imaging data available;
- No history of prior cervical spine surgery.

**FIGURE 1:**
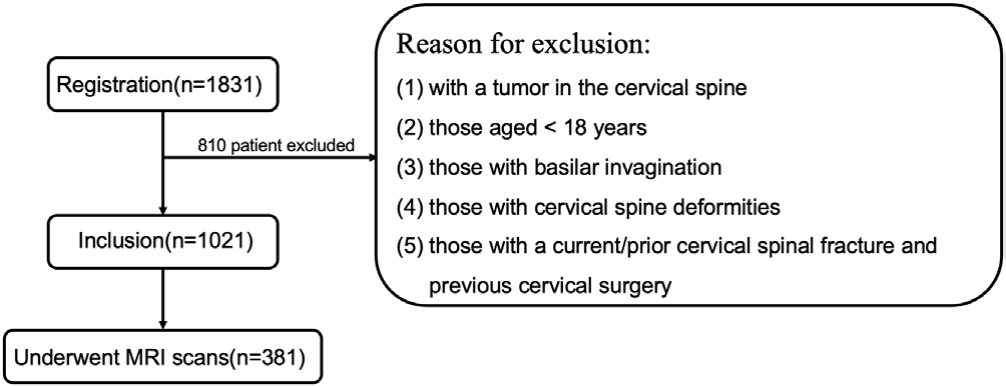
Flow diagram of patient enrolment.

Exclusion criteria were as follows:

- Diagnosis of rheumatoid arthritis;
- Dependence on dialysis;
- Presence of cervical spine tumors;
- Basilar invagination;
- Congenital or acquired cervical spine deformities;
- Current or prior cervical spine fractures;

The flow diagram of patient enrolment is presented in Figure 1. Eventually, 1021 patients met our inclusion criteria. Patients were divided into age groups for analysis: 18–29 years, 30–39 years, 40–49 years, 50–59 years, 60–69 years, 70–79 years, and 80 years or older.

### 2.3 CT Measurements

**FIGURE 2:**
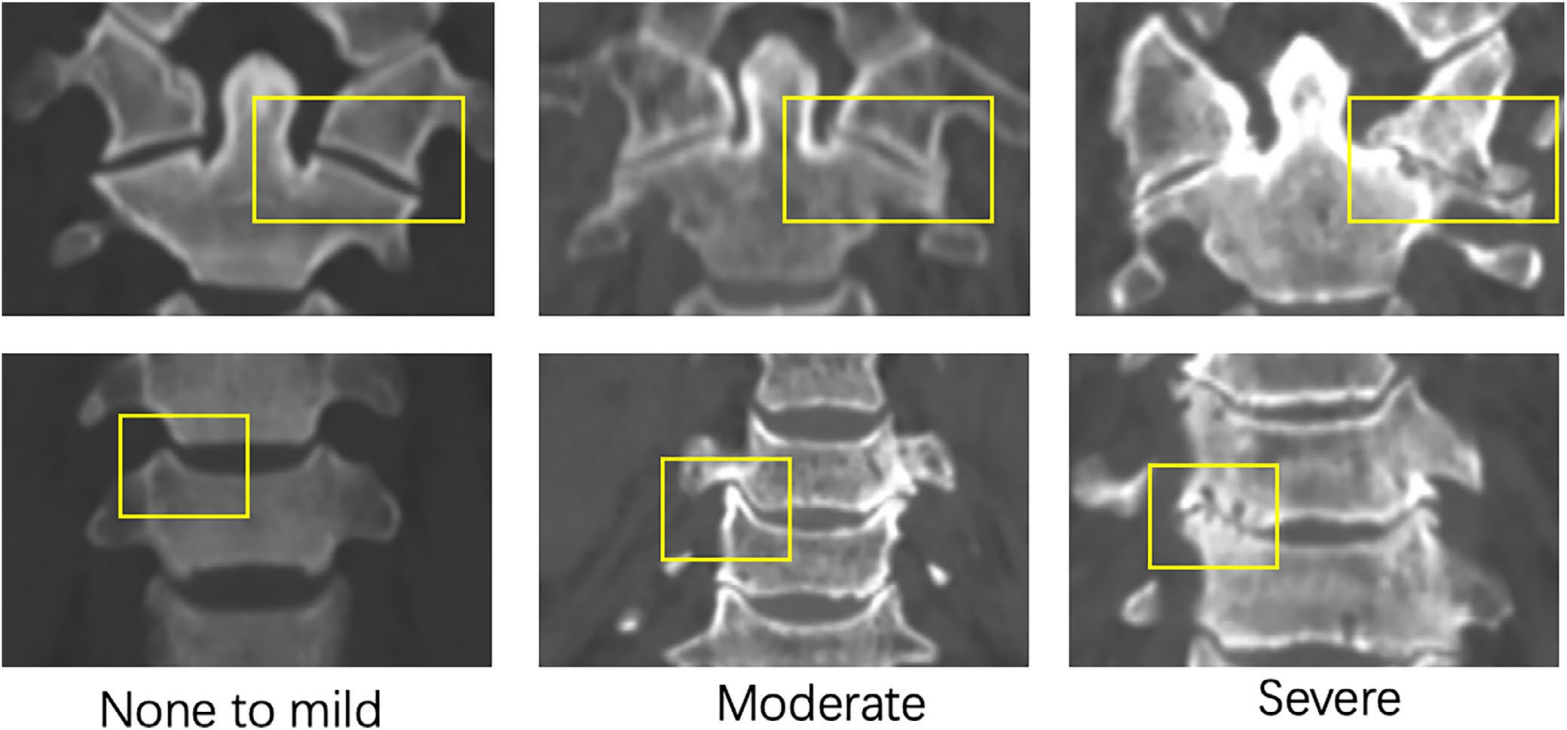
Evaluation of atlantoaxial osteoarthritis and uncovertebral joint degeneration: None to mild (Left) shows normal joint space without osteophyte formation. None to mild (Middle) shows obliterated joint space with osteophyte formation. Severe (Right) shows ankylosis of the joint.

**FIGURE 3:**
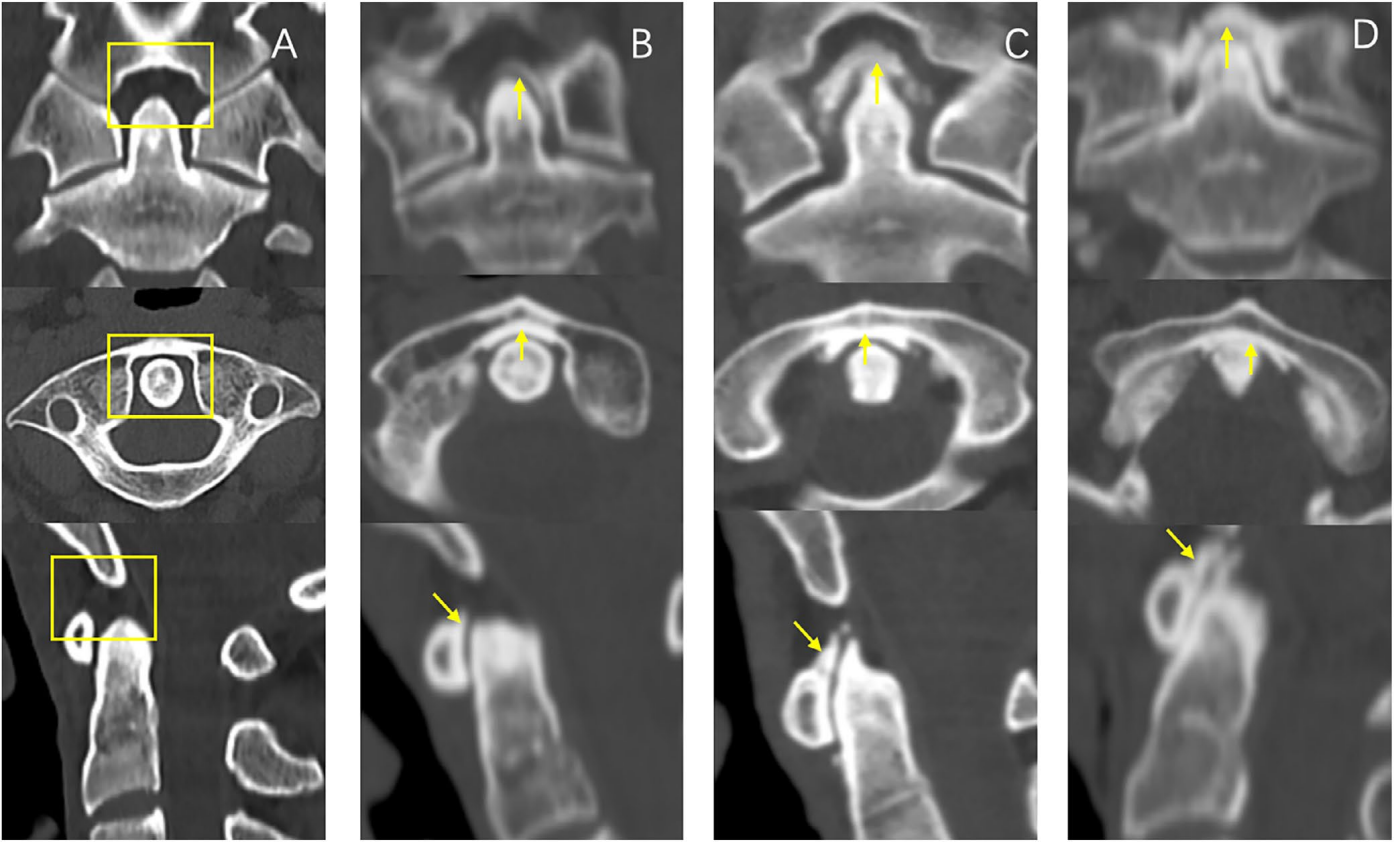
Evaluation of ossification of the atlanto‐occipital ligament. The different grades were based on the length of ossification of interest (XY) with respect to the distance from the posterosuperior rim of the anterior arch of the atlas to the inferior margin of the foramen magnum (AO) on mid‐sagittal cervical spine CT images. (A) Coronal, axial, and sagittal views from a patient with Grade 0 ossification of the atlanto‐occipital ligament. (B) Coronal, axial, and sagittal views from a patient with Grade 1 ossification of the atlanto‐occipital ligament. (C) Coronal, axial, and sagittal views from a patient with Grade 2 ossification of the atlanto‐occipital ligament. (D) Coronal, axial, and sagittal views from a patient with Grade 3 ossification of the atlanto‐occipital ligament.

Cervical spine images were acquired via CT (Siemens, Erlangen, Germany). Degenerative changes were evaluated qualitatively through sagittal and coronal views, focusing on AAOA, uncovertebral joint degeneration, ossification of the atlanto‐occipital ligament, as well as ossification of the posterior and anterior longitudinal ligaments. The severity of AAOA and uncovertebral joint degeneration was assessed using the scoring system from Lakshmanan et al. with severity levels categorized as none, mild, moderate, and severe [21]. Following Betsch et al.'s method [3] the “none–mild” grades were classified as “absence of OA,” while “moderate” and “severe” grades were grouped as “presence of OA” (Table 1, Figure 2). As reported by Liu et al., the degree of atlanto‐occipital ligament ossification was classified into four grades (0–3): Grade 0 indicates no obvious ossification, Grade 1 indicates that the length of ossification (XY) is less than one‐third of the distance from the posterior superior margin of the anterior atlas arch to the inferior margin of the foramen magnum (AO). Grade 2 indicates an ossification length between one‐third and two‐thirds; Grade 3 indicates an ossification of more than two‐thirds length [12] (Figure 3). Two orthopedic surgeons independently evaluated all data from the CT image measurements.

**TABLE 1:** Grading the severity of degenerative changes in lateral atlanto‐axial joints and uncovertebral joints.

1—None‐mild: normal or narrowed joint space with or without minor osteophyte formation
2—Moderate: obliterated joint space with or without osteophyte formation.
3—Severe: ankylosis of the joint with excrescences either in the joint or transverse ligament calcification, or both.

### 2.4 MRI Measurements

**FIGURE 4:**
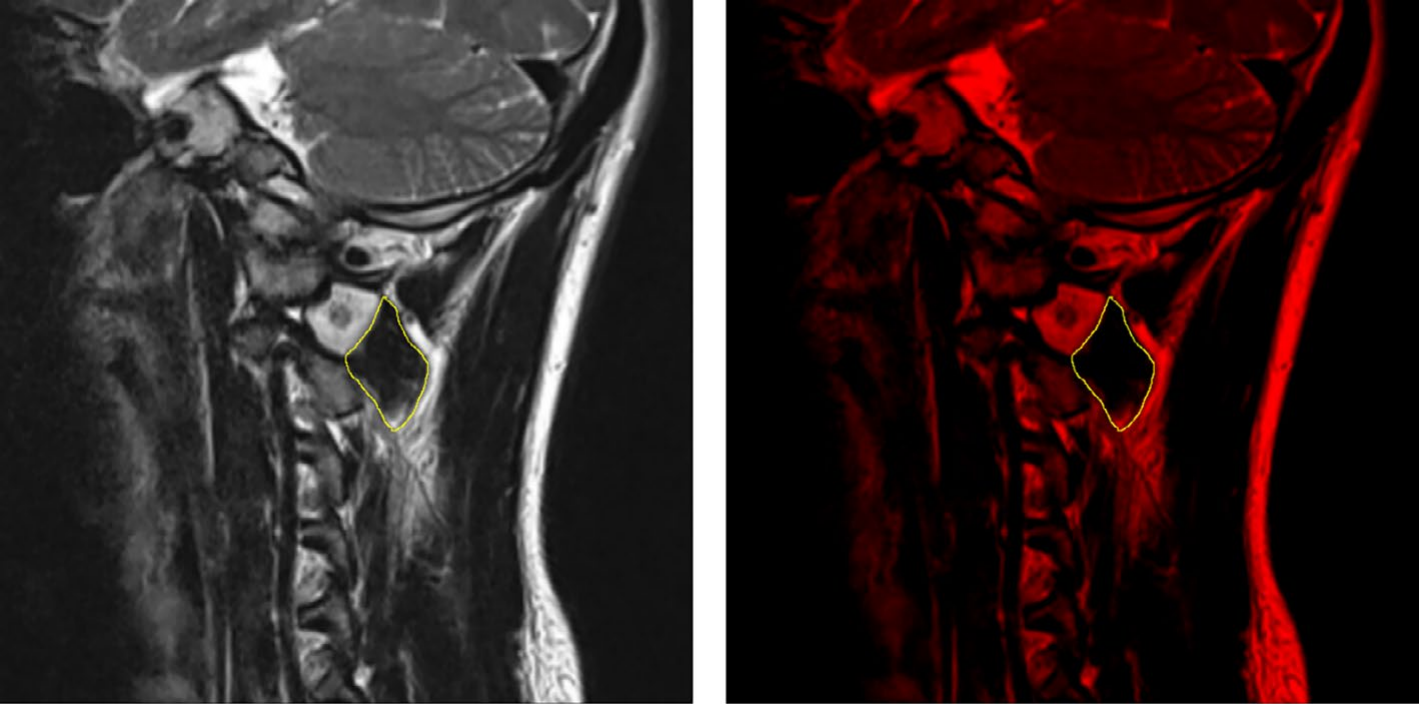
TMCSA and FI ratio of the OCI muscle were measured. (Left) This area within the yellow line represents the multifidus TMCSA. (Right) The red area within the yellow line represents the fatty amount of the multifidus.

There were 381 patients who underwent upper cervical spine MRI scans. Imaging data of these subjects were collected with a 1.5T MRI scanner (Avanto, Erlangen, Germany). The sagittal plane passing through the bilateral C2 intervertebral foramina was selected from T2‐weighted images (T2WI). As illustrated in Figure 4, the OCI muscle boundaries were outlined to define the desired muscle area, and the total muscle cross‐sectional area (TMCSA) was subsequently measured. TMCSA was measured bilaterally using sagittal plane images at the foraminal plane for both the left and right OCI muscles. The final TMCSA value represents the sum of the bilateral muscle areas, ensuring consistency with previously validated methods for assessing deep cervical muscle morphology. The measurement was performed at a standardized anatomical level to ensure accuracy and reproducibility. The sagittal plane was chosen to provide a longitudinal representation of the OCI muscle, allowing for a precise assessment of muscle morphology and fatty infiltration. The measurement was conducted at the foraminal plane, ensuring consistent anatomical positioning across all subjects and minimizing measurement variability. This standardized approach allows for better comparability of muscle size between individuals. Pseudocoloring techniques were applied to highlight adipose tissue as red pixels. The red‐highlighted area within the muscle, termed the fat cross‐sectional area (FCSA), was then quantified using image‐based proportion calculations. The functional muscle cross‐sectional area (FMCSA) was determined by subtracting the fat cross‐sectional area (FCSA) from the total muscle cross‐sectional area (TMCSA). The final TMCSA, FCSA, FMCSA values were calculated as the sum of left and right OCI muscle areas. The degree of fat infiltration (FI) was expressed as the FI ratio, calculated by dividing FCSA by TMCSA. Based on quartiles, FI was categorized into three grades: mild (8.51%–18.49%), moderate (18.67%–31.56%), and severe (31.88%–46.22%). Two orthopedic surgeons independently evaluated all data from the MRI image measurements. To evaluate potential asymmetry between the left and right OCI muscles, a paired‐sample t‐test was conducted to compare the functional muscle cross‐sectional area (FMCSA) bilaterally in AAOA patients.

### 2.5 Statistical Analysis

All statistical analyses were conducted using SPSS statistical software (version 23.0, SPSS Inc., Chicago, IL, USA). Continuous variables are expressed as mean ± standard deviation or median with interquartile range, while categorical variables are reported as absolute numbers and corresponding percentages. Chi‐square tests were conducted to compare the distribution of age, sex, presence of atlanto‐occipital ligament ossification, uncovertebral joint degeneration, anterior longitudinal ligament ossification, and posterior longitudinal ligament ossification between AAOA patients and non‐AAOA patients. Differences in TMCSA, FCSA, FMCSA, and FI between AAOA and non‐AAOA patients were analyzed using *t*‐tests. Before conducting the multivariate logistic regression analysis, we assessed potential collinearity among the independent variables. Variance inflation factor (VIF) and tolerance values were calculated for each predictor variable. A VIF threshold of 5 and a tolerance threshold of 0.2 were used to identify significant multicollinearity concerns. If any variables exceeded these thresholds, they were considered for removal or adjustment to improve model stability. Variables demonstrating significant differences in the univariate regression analysis were incorporated into a multivariate logistic regression model to determine independent factors associated with AAOA. The intra‐class correlation (ICC) was used to assess interobserver reliability. An ICC value of < 0.5 indicates poor reliability, 0.5–0.8 indicates moderate reliability, and > 0.8 indicates good reliability.

An ordinal logistic regression analysis was performed to assess the relationship between AAOA severity (none to mild, moderate, severe) and potential risk factors, including muscle fatty infiltration (FI), uncovertebral joint degeneration, ligament ossification, age, and gender. The proportional odds assumption was tested, and odds ratios (OR) with 95% confidence intervals (CI) were reported.

## 3 Results

### 3.1 Demographic Data

The age distribution of the 1266 patients was recorded, with ages in the cohort ranging from 18 to 98 years (50.18 ± 17.23 years). This study included 606 men (60.6%) and women (59.4%). Among patients with AAOA, the average age was 75.1 ± 9.5 years. The gender distribution comprised 39.3% men and 60.7% women (Table 2).

**TABLE 2:** Summary of patient demographic data.

Characteristics		Total (*n* = 1021)	Without AAOA (*n* = 903)	With AAOA (*n* = 118)	*p*
Age (years)		50.18 ± 17.23	47.47 ± 15.92	70.96 ± 11.91	< 0.001
Sex	Male	606(59.4)	553 (51.2)	53 (44.9)	
	Female	415 (40.6)	350 (38.8)	65 (55.1)	< 0.001
Ossification of the inter‐atlanto‐occipital ligament (*n*, %)		244 (23.9)	188 (20.9)	56 (47.5)	< 0.001
Uncovertebral joint degeneration (*n*, %)		209 (20.5)	149 (16.5)	60 (50.8)	< 0.001
Anterior longitudinal ligament ossification (*n*, %)		169 (16.6)	135 (15.0)	34 (28.8)	< 0.001
Posterior longitudinal ligament ossification (*n*, %)		121 (19.5)	98 (10.9)	23 (19.5)	< 0.001

**FIGURE 5:**
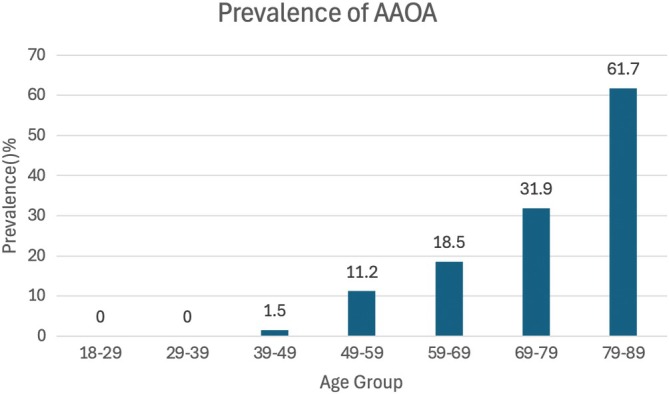
Prevalence of atlantoaxial osteoarthritis among the age groups. Overall, atlantoaxial osteoarthritis was identified in 11.5% of the cases (moderate, 8.8%; severe, 2.7%). The prevalence was significantly higher in people over 50 years of age than in people under 50 years of age (*X* ^2^ = 112.27, *p* < 0.001).

Approximately 11.5% of patients had AAOA, and their grade distribution was as follows: none‐to‐mild, 88.4%; moderate, 8.8%; and severe, 2.7%. In this study, the prevalence of AAOA was about 0.59% in individuals under 50 years old. This rate rose to 11.2% in those in their fifth decade and reached 31.9% in individuals aged 80 and older (Figure 5). Ossification of the atlanto‐occipital ligament was observed in 23.9% of patients. The distribution of grades was as follows: Grade 0, 75.9%; Grade 1, 8.7%; Grade 2, 6.0%; and Grade 3, 9.2% (Table 2). Uncovertebral joint degeneration was found in 20.5% of patients. The distribution by grade was as follows: none‐to‐mild, 79.4%; moderate, 15.4%; and severe, 5.1% (Table 2).

The overall prevalence rates of anterior longitudinal ligament ossification and posterior longitudinal ligament ossification were 16.6% and 11.9%, respectively (Table 2). When we included patients diagnosed with AAOA, with 55.1% of them being female, the prevalence rates of ossification of the atlanto‐occipital ligament, uncovertebral joint degeneration, anterior longitudinal ligament ossification, and posterior longitudinal ligament ossification were 47.5%, 50.8%, 28.8%, and 11.9%, respectively (Table 2). Chi‐square tests revealed significant differences in the distribution of these factors between patients with AAOA and those without AAOA (Table 2).

### 3.2 Ossification of the Atlanto‐Occipital Ligament and Uncovertebral Joint Degeneration Are Potential Risk Factors

In the univariate analysis, the factors listed below were found to be independently and significantly associated with AAOA (Table 3), age in the fifth decade or older (OR = 45.684, *p* < 0.001), female sex (OR = 1.938, *p* < 0.001), anterior longitudinal ligament ossification (OR = 2.303, *p* < 0.001), posterior longitudinal ligament ossification (OR = 1.989, *p* = 0.007), ossification of the atlanto‐occipital ligament (using Grade 0 as the reference; Grade 1: OR = 2.92, *p* < 0.001; Grade 2: OR = 3.43, *p* < 0.001; Grade 3: OR = 3.94, *p* < 0.001), uncovertebral joint degeneration of the lower cervical spine(using Grade 0 as the reference; Grade 1: OR = 3.19, *p* < 0.001; Grade 2: OR = 16.37, *p* < 0.001).

**TABLE 3:** Univariate logistic regression analysis of risk factors and atlantoaxial osteoarthritis in the overall study population.

Characteristics		OR	95% CI	*p*
Age in the fifth decades or older	Without	Ref	Ref	
	With	45.68	14.41–144.81	< 0.001
Sex	Male	Ref	Ref	
	Female	1.94	1.32–2.85	< 0.001
Ossification of the inter‐atlanto‐occipital ligament (%)	Grade 0	Ref	Ref	< 0.001
	Grade 1	2.915	1.64–5.20	< 0.001
	Grade 2	3.426	1.79–6.57	< 0.001
	Grade 3	3.943	2.32–6.71	< 0.001
Uncovertebral joint degeneration (%)	None to mild	Ref	Ref	< 0.001
	Moderate	3.19	1.99–5.14	< 0.001
	Severe	16.37	8.90–30.10	< 0.001
Anterior longitudinal ligament ossification (%)	Without	Ref	Ref	
	With	2.30	1.49–3.57	< 0.001
Posterior longitudinal ligament ossification (%)	Without	Ref	Ref	
	With	1.989	1.21–3.28	0.007

The results of the multivariate analysis for identifying independent risk factors for AAOA are presented in Table 4. The factors below were identified as independently and significantly associated with AAOA: age in the fifth decade or older (OR = 27.723, *p* < 0.001), ossification of the atlanto‐occipital ligament (OR = 1.217, *p* = 0.035), women's sex (OR = 2.630, *p* < 0.001), and uncovertebral joint degeneration of the lower cervical spine (OR = 2.364, *p* < 0.001).

**TABLE 4:** Multivariate logistic regression model for the development of atlantoaxial osteoarthritis.

Characteristics		Odds ratio	95% CI	*p*
Age in the fifth decade or older		30.48	9.37–99.04	< 0.001
Sex	Male	Ref	Ref	
	Female	2.54	1.65–3.90	< 0.001
Ossification of the inter‐atlanto‐occipital ligament	Without	Ref	Ref	
	With	1.59	1.03–2.45	0.037
Uncovertebral joint degeneration	Without	Ref	Ref	
	With	2.38	1.53–3.68	< 0.001
Anterior longitudinal ligament ossification	Without	Ref	Ref	
	With	0.94	0.57–1.56	0.944
Posterior longitudinal ligament ossification	Without	Ref	Ref	
	With	1.60	0.89–2.88	1.601

### 3.3 Severe Fatty Infiltration of the OCI Muscles is Another Potential Risk Factor

**FIGURE 6:**
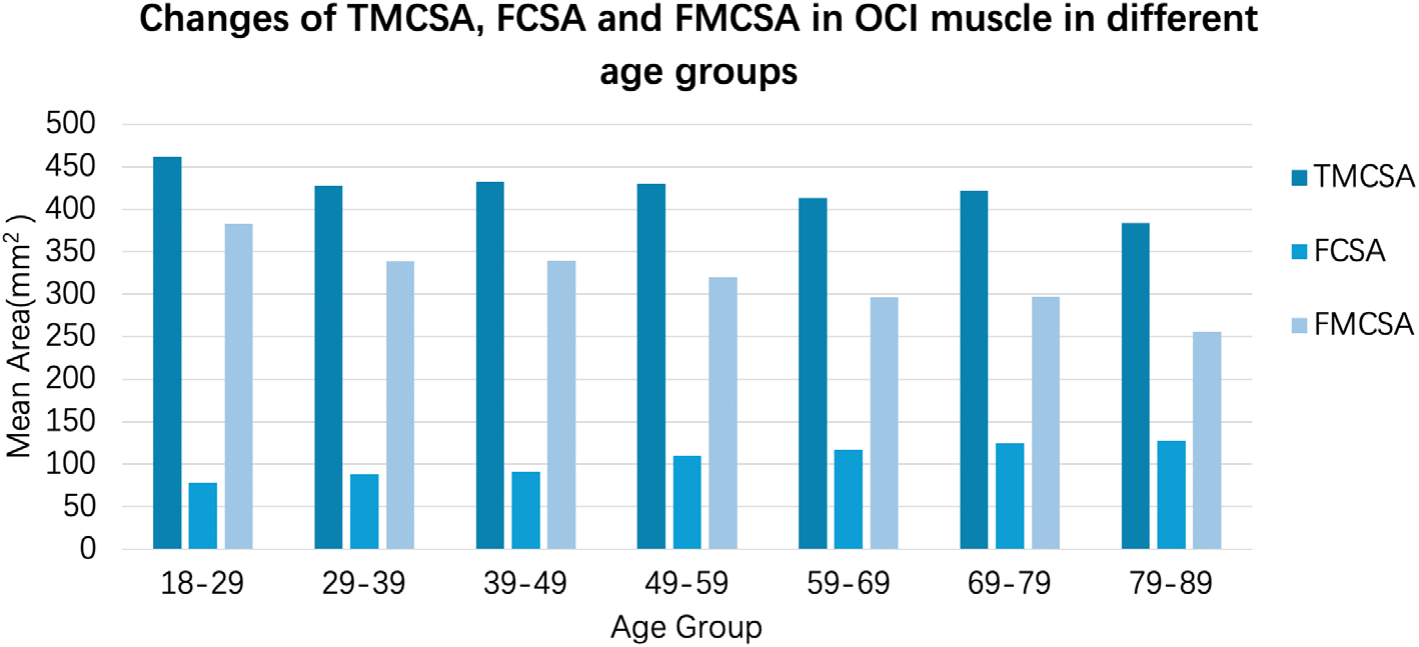
The relation between age and TMCSA, FCSA, FMCSA. We found that TMCSA had no significant correlation with age (*R* ^2^ = −0.71, *p* = 0.204), while FCSA, FMCSA, and age had significant correlation (FCSA: *R* ^2^ = 0.34, *p* < 0.001; FMCSA: *R* ^2^ = −0.23, *p* < 0.001).

**FIGURE 7:**
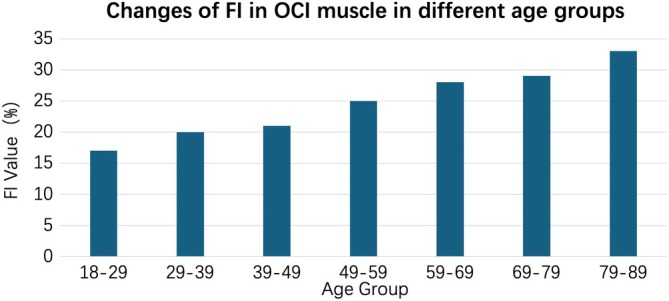
The relation between age and FI ratio. There was a significant correlation between FI and age (*R* ^2^ = 0.51, *p* < 0.001).

The mean values of TMCSA, FCSA, FMCSA, and FI of the OCI muscles in patients who underwent MRI were 424.70% ± 135.98%, 107.94% ± 52.20%, 316.75% ± 108.99%, and 25.42% ± 8.50%, respectively. Figures 6 and 7 illustrate how these values change with age. In AAOA patients, the functional muscle cross‐sectional area (FMCSA) was 138.89 ± 50.56 mm^2^ on the right side and 135.66 ± 48.80 mm^2^ on the left side. A paired‐sample *t*‐test indicated no significant difference between the two sides (*t* = 1.02, *p* = 0.311), suggesting that muscle asymmetry was not a contributing factor in AAOA. When comparing AAOA patients to non‐AAOA patients, there was no significant difference in TMCSA (*t* = 1.22, *p* = 0.225). However, as shown in Table 5, significant differences were found in FCSA (*t* = −4.64, *p* < 0.001), FMCSA (*t* = 3.74, *p* < 0.001), and FI (*t* = −9.74, *p* < 0.001), indicating a potential relationship between these variables and the presence of AAOA. In multivariate logistic regression analysis (Table 6), it was found that greater fatty infiltration was also significantly associated with an increased risk of AAOA (OR = 3.52, *p* < 0.001).

**TABLE 5:** Comparison of the mean FCSA, FMCSA, TMCSA and FI ratio in AAOA patients and non‐AAOA patients.

	Without AAOA	AAOA	*t*	*p*
TMCSA (mm^2^)	428.66	406.70	1.22	0.225
FCSA (mm^2^)	102.27	133.67	−4.64	< 0.001
FMCSA (mm^2^)	326.39	273.04	3.74	< 0.001
FI (%)	23.86	32.49	−9.74	< 0.001

**TABLE 6:** Multivariate logistic regression model for the development of atlantoaxial osteoarthritis including FI level of the OCI muscle.

Characteristics		Odds ratio	95% CI	*p*
Age in the fifth decade or older	Without	Ref	Ref	
	With	27.88	3.76–206.58	< 0.001
Sex	Male	Ref	Ref	
	Female	2.10	1.16–3.80	0.014
FI level of the OCI muscle	Grade 0	Ref	Ref	< 0.001
	Grade 1	5.32	1.21–23.45	< 0.001
	Grade 2	16.78	3.77–74.72	< 0.001

## 4 Factors Associated With AAOA Severity: Insights From Ordinal Logistic Regression Analysis

Ordinal logistic regression analysis revealed that muscle fatty infiltration (FI), uncovertebral joint degeneration, age, and gender were significantly associated with AAOA severity. Compared to patients with severe FI (reference category, OR = 1.00), those with mild FI (OR = 0.0739, *p* < 0.001) and moderate FI (OR = 0.3546, *p* < 0.001) had significantly lower odds of severe AAOA progression, suggesting a dose–response relationship between FI severity and AAOA. Similarly, uncovertebral joint degeneration was significantly associated with AAOA severity, with patients exhibiting mild degeneration (Grade 0, OR = 0.3822, *p* < 0.05) and moderate degeneration (Grade 1, OR = 0.3647, *p* < 0.05) having significantly lower odds of severe AAOA compared to those with severe degeneration (Grade 2, reference category, OR = 1.00). Older age (*p* = 0.002, OR = 0.0399) and male gender (*p* = 0.040, OR = 0.5370) were also associated with a lower likelihood of severe AAOA, indicating potential protective effects. In contrast, different degrees of ligament ossification did not show a statistically significant association with AAOA severity (*p* > 0.05). Full regression results, including odds ratios (OR), confidence intervals (CI), and statistical significance, are provided in Table S1.

### 4.1 Collinearity Diagnostics

The collinearity analysis indicated that all independent variables had a VIF < 5 and tolerance values > 0.2, confirming that multicollinearity was not a concern in our regression model. This ensures that the estimated regression coefficients remain stable and reliable. Full collinearity diagnostic results are provided in Table S2.

### 4.2 Interobserver Reliability Results

This study assessed interobserver reliability for the following parameters: the severity of AAOA, the severity of uncovertebral joint degeneration, the severity of atlanto‐occipital ligament ossification, the presence of ossification in the anterior longitudinal ligament and posterior longitudinal ligament, as well as the TMCSA, FCSA, FMCSA, and FI of the OCI muscle. The results showed that the interobserver ICC values for these nine parameters were 0.983, 0.921, 0.986, 0.980, 0.965, 0.925, 0.969, 0.966, and 0.955, respectively, all exceeding 0.8, indicating excellent interobserver reliability.

## 5 Discussion

This study found that the prevalence of AAOA in our cohort is 11.5%. Through multivariable logistic regression analysis, we identified significant risk factors for AAOA, including age in the fifth decade or older, ossification of the inter‐atlanto‐occipital ligament, female sex, and uncovertebral joint degeneration. Severe fatty infiltration of the OC muscles may also be another potential risk factor.

### 5.1 Age and Sex

Several studies to date have reported an increased prevalence of AAOA with advancing age [3, 4, 22]. Betsch et al. [3] reported a prevalence of 9.5%–9.8% for AAOA in the general population, while noting that the prevalence is less than 10% among individuals aged 18–67 years, but increases significantly to 35%–45% in those aged ≥ 88 years, which is very close to our findings. However, Yuma Suga et al. [7] reported a prevalence of only 2.5% for AAOA, which is significantly lower than our results. This discrepancy may be attributed to the higher proportion of elderly individuals in our cohort.

Our study found a significant association between female sex and an increased risk of atlantoaxial osteoarthritis (AAOA), aligning with the findings of Yuma Suga et al. [7] Women have already been proven to be at higher risk for various cervical degenerative changes, which may be related to their unique anatomical structures and hormonal factors [23, 24, 25]. These results suggest that elderly women may benefit from early screening for AAOA to facilitate timely intervention.

### 5.2 Uncovertebral Joint Degeneration

Our study identified a significant correlation between uncovertebral joint degeneration and the presence of atlantoaxial osteoarthritis (AAOA), with more severe uncovertebral joint degeneration showing a stronger association with AAOA. The uncovertebral joints play a vital role in facilitating cervical spine movement and maintaining spinal alignment [26]. As these joints degenerate, the resultant loss of stability and range of motion may increase biomechanical stress on adjacent cervical segments, including the atlantoaxial joint [27].

This increased stress could lead to compensatory changes in the atlantoaxial joint, accelerating its degeneration and contributing to the development of AAOA [6]. Our findings suggest that the severity of uncovertebral joint degeneration may serve as a key factor in the progression of AAOA, as the most advanced stages of uncovertebral degeneration were associated with the highest prevalence of AAOA in our cohort. Therefore, uncovertebral joint degeneration, particularly in its more advanced stages, should be considered a critical risk factor for AAOA. Our findings support the hypothesis that early uncovertebral joint degeneration may be a precursor to AAOA, warranting further investigation into whether early intervention—such as posture correction or cervical stabilization exercises—can mitigate disease progression.

### 5.3 Ossification of the Atlanto‐Occipital Ligament

Ossification of the inter‐atlanto‐occipital ligament was observed in 23.9% of patients, with higher ossification grades correlating with increased AAOA prevalence. However, ordinal logistic regression analysis suggests that ossification of the inter‐atlanto‐occipital ligament may not be a significant factor in AAOA progression. Unlike previous assumptions that ligament ossification plays a primary role in disease advancement, our findings indicate that its presence does not independently predict AAOA progression. This result underscores the complexity of AAOA pathogenesis and suggests that other degenerative and biomechanical factors may contribute more significantly to disease progression [12, 28, 29].

Biomechanical studies have demonstrated that ligament ossification itself does not necessarily lead to joint degeneration but rather reflects underlying systemic or localized degenerative changes [29]. Furthermore, prior investigations into the craniocervical junction indicate that mechanical stress redistribution, facet joint degeneration, and asymmetric loading may be more significant contributors to AAOA progression than ligament ossification alone [28, 30].

Recent clinical research has shown that surgical removal of ligament ossification does not consistently improve clinical outcomes, as its impact on symptoms remains uncertain [30]. Instead, conservative management strategies, such as cervical mobility training and postural correction, may help patients maintain cervical spine function and reduce compensatory mechanical stress [15, 30]. Given these findings, future studies should explore whether targeted rehabilitation programs and biomechanical assessments can help prevent further cervical degeneration in individuals with ligament ossification.

### 5.4 Fatty Infiltration of the OCI Muscles

Our study revealed a significant association between fatty infiltration (FI) of the obliquus capitis inferior (OCI) muscle and the presence of atlantoaxial osteoarthritis (AAOA). The OCI muscle plays a crucial role in stabilizing the atlantoaxial joint by facilitating head rotation and maintaining the alignment of the craniocervical junction [31]. As the muscle undergoes fatty infiltration, its functional capacity diminishes, potentially compromising the stability of the atlantoaxial joint and leading to increased mechanical stress on the joint structures [32].

The mechanism by which OCI fatty infiltration contributes to AAOA may be linked to joint instability caused by weakened muscle support. As the OCI muscle loses strength due to the replacement of muscle fibers with fat, it may no longer be able to effectively stabilize the joint, which could lead to abnormal movement patterns, joint misalignment, and accelerated degenerative changes in the atlantoaxial joint [30]. This is consistent with previous studies that have demonstrated the role of muscle degeneration in the progression of other spinal degenerative conditions, such as cervical spondylosis and post‐surgical complications [30, 32, 33, 34].

Our findings indicate that severe OCI fatty infiltration may act as a key risk factor for the development and progression of AAOA. The relationship between more pronounced FI and the presence of AAOA suggests that the degree of muscle degeneration could be directly related to the severity of joint degeneration. This highlights the importance of considering muscle health, particularly in the suboccipital region, in the early identification and management of AAOA.

These findings highlight the need for future research into rehabilitation strategies, such as targeted cervical muscle strengthening, to determine whether improving OCI muscle function can reduce AAOA risk.

### 5.5 Clinical Implications and Future Directions

While this study identifies important risk factors, the clinical application of these findings requires further exploration. Currently, there is no strong evidence supporting surgical intervention for ligament ossification in AAOA prevention, and the efficacy of cervical muscle training for joint stabilization remains uncertain. Future prospective studies should evaluate whether early rehabilitation or lifestyle modifications can alter disease progression. Additionally, biomechanical modeling studies suggest that changes in cervical alignment and joint loading play a crucial role in AAOA development, emphasizing the need for further analysis of load distribution and compensatory mechanisms [28, 29].

Furthermore, rehabilitation techniques targeting muscle imbalance and posture correction may help reduce asymmetric mechanical stress on the atlantoaxial joint, potentially slowing AAOA progression [30]. Clinical trials investigating the efficacy of physical therapy interventions, bracing, and lifestyle modifications could provide further insights into non‐surgical treatment approaches. Future research should also consider integrating longitudinal cohort studies to assess how modifying these risk factors influences AAOA incidence and severity over time.

In future studies, targeted rehabilitation programs may focus on deep cervical flexor training, proprioceptive exercises, and postural retraining, which have been shown to improve cervical spine stability and reduce abnormal joint stress in other degenerative conditions. Bracing strategies designed to correct forward head posture or reduce upper cervical hypermobility may also be explored, particularly in early‐stage patients with biomechanical imbalance. Additionally, lifestyle interventions such as ergonomic modifications, avoidance of prolonged neck flexion, and promotion of regular low‐impact exercise (e.g., swimming or yoga) may help mitigate the progression of AAOA. These hypotheses could be evaluated through prospective interventional studies or randomized controlled trials assessing functional outcomes, radiological progression, and patient‐reported symptom scores over time.

### 5.6 Limitations and Strengths

Several important limitations should be considered when interpreting our findings. First, as a single‐center study conducted at a tertiary hospital in Wenzhou, China, our results may not fully represent the broader population due to potential geographical variations in lifestyle factors and healthcare access patterns. Second, while our study spanned a 10‐year period (2014–2024), we were unable to systematically account for evolving lifestyle trends such as increased smartphone usage and changes in occupational ergonomics that may influence cervical spine degeneration. Third, our retrospective design limited our ability to collect detailed information on potentially important confounding variables, including specific occupational histories (particularly for jobs requiring prolonged head‐down postures), genetic predispositions, and socioeconomic factors. These limitations highlight the need for future prospective, multicenter studies that incorporate comprehensive lifestyle assessments and genetic profiling to better understand the complex interplay of risk factors for atlantoaxial osteoarthritis.

This study has several strengths. First, it utilizes a well‐characterized retrospective cohort with comprehensive imaging data, including both CT and MRI. Second, the study applies a multivariate approach integrating ligament ossification, joint degeneration, and muscle morphology, allowing for a holistic understanding of AAOA pathogenesis. Third, we included ordinal logistic regression to explore not only risk association but also progression severity. These methodological choices enhance the robustness and clinical relevance of our findings.

## 6 Conclusion

This study provides a comprehensive analysis of the risk factors associated with atlantoaxial osteoarthritis (AAOA) in a clinical cohort from Eastern China, revealing a prevalence of 11.5% among adults. Our findings indicate that age over 50, female sex, uncovertebral joint degeneration, inter‐atlanto‐occipital ligament ossification, and severe fatty infiltration of the OCI muscle are independent risk factors for AAOA. While surgical interventions remain controversial, early screening and targeted rehabilitation may offer potential preventive strategies. Further research is warranted to explore whether non‐surgical interventions can effectively reduce the incidence or severity of AAOA and improve patient outcomes.

## Author Contributions

Shuqing Jin, Shuhao Zhang, and Yuxiao Zhu contributed equally to this work and are recognized as co‐first authors. Xiangyang Wang served as the corresponding author, overseeing the study design, data analysis, and manuscript preparation. Shuqing Jin and Shuhao Zhang participated in the study design and conducted data collection. Yuxiao Zhu performed the statistical analysis and drafted the manuscript. Yan Chen, Yiting Tu, and Yurui Wu contributed to image analysis and data interpretation. Siyu Hu and Chen Xiang assisted in manuscript revision and ensured the accuracy of clinical data. All authors reviewed and approved the final manuscript.

## Disclosure

All authors listed meet the authorship criteria according to the latest guidelines of the International Committee of Medical Journal Editors, and all authors are in agreement with the manuscript.

## Ethics Statement

Institutional review board approval for this study (MR‐33‐25‐000548) was obtained from The Second Affiliated Hospital and Yuying Children's Hospital of Wenzhou Medical University.

## Conflicts of Interest

The authors declare no conflicts of interest.

## Supporting information


**Data S1.** Supporting Information.
