# Supplementary material for: Multivariate Analysis of Risk Factors for Atlantoaxial Osteoarthritis: A Retrospective Cohort Study on Ligament Ossification, Joint Degeneration, and Muscle Fatty Infiltration
Source: Orthop Surg. 2025 Sep 1;17(10):2851–61. doi: 10.1111/os.70125 (PMC12497544; doi:10.1111/os.70125)
Supplement: Supplementary file 1 — Data S1. Supporting Information. [file OS-17-2851-s001.docx]

Table S1. Ordinal Logistic Regression Results for the Association Between AAOA Severity, Ligament Ossification, Joint Degeneration, and Muscle Fatty Infiltration

| Characteristics |  | Estimate (B) | OR  (Exp(B)) | CI 95% | P-value |
| --- | --- | --- | --- | --- | --- |
| Age in the fifth decade or older |  | -3.23 | 0.04 | -5.24 to 1.21 | 0.002 |
| Sex: | Male | -0.62 | 0.54 | -1.21 to 0.03 | 0.04 |
|  | Female | ref | 1.00 | ref |  |
| Ossification of the inter-atlanto-occipital ligament | Grade0 | -0.22 | 0.81 | -0.97 to 0.54 | 0.58 |
|  | Grade1 | -0.04 | 0.96 | -1.04 to 0.95 | 0.93 |
|  | Grade2 | -0.68 | 0.51 | -1.80 to 0.45 | 0.24 |
|  | Grade3 | ref | 1.00 | ref |  |
| Uncovertebral joint degeneration | None to mild | -0.96 | 0.38 | -1.84 to 0.08 | 0.032 |
|  | Moderate | -1.01 | 0.36 | -1.96 to 0.05 | 0.039 |
|  | Severe | ref | 1.00 | ref |  |
|  | Grade0 | -2.61 | 0.07 | -4.13 to 1.08 | <0.001 |
| FI level of the OCI muscle | Grade1 | -1.04 | 0.35 | -1.65 to 0.42 | <0.001 |
|  | Grade2 | red | 1.00 | ref |  |

| Characteristics | Tol | VIF |
| --- | --- | --- |
| Age in the fifth decade or older | 0.799 | 1.25 |
| Female sex | 0.99 | 1.01 |
|  |  |  |
| With ossification of the inter-atlanto-occipital ligament | 0.88 | 1.13 |
| With uncovertebral joint degeneration | 0.85 | 1.12 |
| With anterior longitudinal ligament ossification | 0.82 | 1.21 |
| With posterior longitudinal ligament ossification | 0.87 | 1.14 |
| With different FI level of the OCI muscle | 0.82 | 1.22 |

Table S2. Collinearity Assessment of Independent Variables
